# Combating Healthcare-Associated Infections in Modern Hospitals: Nanotechnology-Based Approaches in the Era of Antimicrobial Resistance

**DOI:** 10.3390/nano15181405

**Published:** 2025-09-12

**Authors:** Federica Paladini, Fabiana D’Urso, Francesco Broccolo, Mauro Pollini

**Affiliations:** Department of Experimental Medicine, University of Salento, 73100 Lecce, Italy; fabiana.durso@unisalento.it (F.D.); francesco.broccolo@unisalento.it (F.B.)

**Keywords:** antimicrobial resistance, healthcare-associated infections, silver nanoparticles, nanotechnology

## Abstract

Healthcare-associated infections (HAIs) represent one of the most persistent challenges in modern healthcare delivery, affecting millions of patients worldwide and imposing substantial clinical and economic burdens on healthcare systems. The emergence of antimicrobial resistance (AMR) has further complicated infection management, creating an urgent need for innovative therapeutic and preventive strategies. Current strategies for combating AMR in hospital settings encompass comprehensive infection prevention and control measures, antimicrobial stewardship programs, enhanced environmental cleaning protocols and innovative surface modification technologies. Nanotechnology has emerged as a valuable approach to address the limitations of conventional antimicrobial strategies. Various nanomaterial categories offer innovative platforms for developing novel treatment strategies and for providing advantages including reduced toxicity through lower dosage requirements, diminished resistance development potential, and enhanced antibacterial effects through combined action mechanisms. Particularly, metal-based nanoparticles and their oxides demonstrate exceptional antimicrobial properties through multiple mechanisms including membrane damage, protein binding and reactive oxygen species generation. This comprehensive review examines the current landscape of hospital-acquired infections, the growing threat of antimicrobial resistance, and the promising role of nanotechnology-based solutions, with particular emphasis on silver nanoparticles as innovative tool for HAI control in clinical settings. Recent advances in nanotechnology-enabled antimicrobial coatings are assessed along with their clinical translation in hospital settings, identifying key barriers concerning material durability, safety profiles, and regulatory pathways.

## 1. The Problem of Hospital-Acquired Infections

### 1.1. Definition and Epidemiological Impact

Healthcare-associated infections, commonly referred to as nosocomial infections or hospital-acquired infections (HAIs), constitute a fundamental challenge in contemporary medical practice [1]. The etymology of “nosocomial” traces back to ancient Greek terminology, where “nosus” (disease) and “komeion” (to care for) combine to describe infections arising within care environments [2].

Healthcare-associated infections are characterized as infectious processes that develop in patients during their stay in medical facilities, typically emerging 48 h post-admission when no infection was present or incubating upon hospital entry [3]. These infections, frequently involving multidrug-resistant pathogens, contribute significantly to patient morbidity, extended hospitalization periods, increased mortality rates, and substantial healthcare expenditures.

### 1.2. Risk Factors and Transmission Mechanisms

Long-term care facility residents face heightened vulnerability to HAI acquisition due to multiple risk factors, such as advanced patient age, underlying medical conditions, compromised cognitive and physical functioning, immunosuppressive treatment regimens, and invasive medical device utilization. The close proximity between healthcare personnel and residents, combined with frequent inter-facility transfers, creates optimal conditions for infection transmission and cross-contamination between acute and long-term care environments [4]. The consequences of HAIs encompass prolonged hospital stays, permanent disabilities, and dramatically increased healthcare costs, fundamentally compromising patient safety and care quality across all medical settings [5].

### 1.3. Global Burden and Mortality Statistics

The magnitude of this global healthcare challenge is remarkable [3]. On a worldwide scale, hospital-acquired infections constitute the most frequent adverse healthcare events, affecting hundreds of millions of patients annually and generating substantial morbidity, mortality, and economic burden for healthcare systems globally [6]. Recent data from the 2024 WHO Global Report on Infection Prevention and Control and ECDC’s point prevalence survey in 2023 confirm these alarming trends, with updated figures showing continued high prevalence rates across healthcare facilities worldwide [7,8]. United States data indicates that HAIs represent the most prevalent hospital care complications and rank among the leading ten mortality causes, while European Union statistics demonstrate that 6.5% of acute care patients experience at least one HAI [3]. The European Centre for Disease Prevention and Control reports that approximately 80,000 patients (representing one in eighteen hospital patients) across European healthcare facilities experience healthcare-associated infections daily [3]. Direct mortality attributable to nosocomial infections reaches approximately 37,000 deaths annually within the EU, potentially escalating to 110,000 when including indirect mortality [3].

### 1.4. Pathogen Reservoirs and Transmission Vehicles

Nosocomial pathogens represent the microbial agents responsible for HAI development, characterized by two essential features, namely lethal potential without appropriate treatment and environmental persistence sufficient for transmission occurrence. Hospital environments, particularly high-contact surfaces, experience continuous exposure to potentially pathogenic microorganisms. These contaminated surfaces and inanimate objects, termed fomites, serve as pathogen carriers with transmission potential to humans. Extensive epidemiological research has established fomites as crucial pathogen reservoirs facilitating microbial transmission within hospital settings [5]. Medical textiles represent additional potential transmission vehicles [3].

### 1.5. Device-Associated Infections

Healthcare-associated infections may result from invasive medical procedures, medical device exposure or person-to-person transmission when infection control measures prove inadequate [9]. Medical devices including venous and urinary catheters are extensively utilized in hospital practice, yet their implementation frequently involves complications due to microbial surface adherence and biofilm formation [10]. Venous catheter insertion necessarily breaches skin barriers, permitting skin flora or environmental contaminants to access deeper tissues and potentially cause life-threatening complications such as bloodstream infections [10]. Central line-associated bloodstream infections (CLABSIs) develop when pathogenic microorganisms access the circulatory system through central venous access devices, potentially progressing to sepsis and life-threatening complications [2,11]. Annual occurrences include 80,000 central venous catheter-associated bloodstream infections, primarily within intensive care units, with mortality rates ranging from 12 to 25% [10]. Catheter-associated urinary tract infections (CAUTIs) arise following urinary catheter placement, with infection risk correlating directly with catheterization duration [2,12]. These infections rank among the most prevalent hospital-acquired infections globally, comprising up to 40% of nosocomial infections [10].

### 1.6. Ward-Specific Infection Prevalence

**Figure 1:**
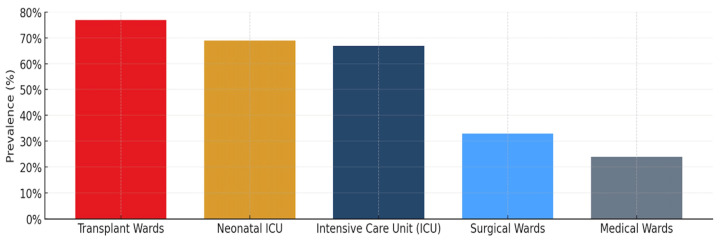
Representation of the healthcare associated infections prevalence by hospital wards. The highest HAI prevalence (77%) corresponds to transplant units, followed by neonatal wards (69%) and intensive care units (68%) [13].

The analysis of healthcare-associated infection (HAI) distribution across different hospital wards reveals significant variations in prevalence rates, reflecting the diverse patient vulnerability and different levels of infectious risk associated with specific types of care. Based on the global meta-analysis data from Raoofi et al. (2023), transplant units exhibit the highest HAI prevalence at 77% (95% CI, 0.38–0.90), followed by neonatal wards at 69% (95% CI, 0.47–0.85) and intensive care units (ICU) at 68% (95% CI, 0.61–0.73) (Figure 1) [13].

### 1.7. Biofilm-Associated Infections and Resistant Pathogens

Surgical site infections (SSIs) can affect multiple tissue layers within surgical wounds, ranging from superficial skin and subcutaneous involvement to deep tissue, organ, or surgical space infections [2,14]. Ventilator-associated pneumonia (VAP) and hospital-acquired pneumonia (HAP) represent severe respiratory complications that significantly extend hospitalization duration and elevate mortality risk [2,15]. Approximately 80% of chronic infections involve biofilm formation. Biofilms comprise microorganism collections organized within extracellular polymeric material matrices, consisting of microbial cells adhering to surfaces and each other, whether living or non-living. Recent research emphasizes that biofilm dispersal mechanisms and metabolic heterogeneity are central to treatment failure, making these infections particularly challenging to eradicate and highlighting the critical need for nanomaterials that can effectively disrupt biofilm matrices [16,17]. The National Institute of Health reports biofilm formation association with 65% of all microbial diseases and 80% of chronic conditions. Biofilm-embedded bacteria survive extended periods on contaminated surfaces despite regular cleaning and disinfection protocols, with room humidity and moist microclimates sustaining biofilm-forming species [18]. Methicillin-resistant *Staphylococcus aureus* (MRSA) infections present particular challenges, extending average hospitalization from 4 to 14 days with additional costs ranging from EUR 10,000 to EUR 36,000 per patient [3].

Predominant nosocomial infection pathogens include S. aureus (including antibiotic-resistant MRSA), *Escherichia coli*, *Enterococcus species*, and *Candida species* [2]. *Carbapenem-resistant Acinetobacter baumannii* represents an urgent public health threat, readily contaminating patient care environments and healthcare provider hands, surviving extended periods on dry surfaces, and spreading through asymptomatic carriers, making hospital outbreak control exceptionally challenging [19].

### 1.8. COVID-19 Pandemic Impact

The COVID-19 pandemic has substantially altered the hospital-acquired infection landscape [5]. Pandemic response measures included enhanced infection prevention and control protocols emphasizing hand hygiene awareness, though isolating hand hygiene effects from concurrent infection control strategies remains challenging [20].

Accumulating evidence suggests increased HAI rates and consumption of antibiotics during the COVID-19 pandemic, amplifying the dimensions of antimicrobial resistance [21,22,23]. Multicenter retrospective analysis of severe COVID-19 patients admitted to eight Italian hospitals revealed that among 774 patients, 46% developed HAIs, with 35% caused by multidrug-resistant bacteria. Ventilator-associated pneumonia (50%), bloodstream infections (34%), and catheter-related bloodstream infections (10%) predominated, with HAIs prolonging mechanical ventilation and hospitalization, while septic shock complications nearly doubled mortality [24].

## 2. Antimicrobial Resistance

### 2.1. Historical Perspective and Emergence

Penicillin’s identification in 1928 and subsequent clinical implementation during the 1940s for treating severe infections represented a revolutionary advancement in medical practice, preserving millions of lives worldwide. Clinical penicillin application began extensively in 1943, yet within one decade, resistance to this antibiotic had developed into a significant therapeutic challenge. This resistance development pattern has been consistently observed with every subsequently approved antimicrobial agent [9,23]. The World Health Organization and national epidemiological surveillance agencies have increasingly highlighted this phenomenon over the past two decades globally. Multiple factors drive this resistance emergence, including antimicrobial overutilization, inappropriate prescribing practices, and widespread agricultural antibiotic application [9].

### 2.2. Current Mortality and Morbidity Burden

Recent evidence from The Lancet (2024) shows that bacterial antimicrobial resistance (AMR) causes over one million deaths annually, with *Staphylococcus aureus* and *Escherichia coli* among the leading contributors; these findings underscore the escalating crisis and the need for effective countermeasures [25,26]. A pivotal recent study demonstrated that antimicrobial resistance (AMR) caused more deaths in 2019 than any other infectious disease, surpassing human immunodeficiency virus (HIV) and malaria mortality [23]. A study published in 2024 revealed that between 1990 and 2021, more than one million people die each year from drug-resistant infections [26,27], with *Escherichia coli* and *Staphylococcus aureus* representing the most lethal pathogens during this timeframe. The 2022 Global Antimicrobial Resistance and Use Surveillance System (GLASS) findings indicate that 35% of *S. aureus* isolates demonstrate methicillin resistance, while 42% of *E. coli* strains exhibit third-generation cephalosporin resistance and diminished susceptibility to standard antimicrobials including ampicillin, co-trimoxazole, and fluoroquinolones, thereby complicating effective treatment of common infections [27]. Across European acute-care hospitals, ICU wards carry the highest burden of HAI and antimicrobial use (HAI prevalence 20.5%; antimicrobial use 59.5%, 2022–2023). In parallel, the EU/EEA incidence of bloodstream infections due to carbapenem-resistant *Klebsiella pneumoniae* increased by 57.5% from 2019 to 2023, reaching 3.97 per 100,000 population with a statistically significant rising trend. Together, these signals indicate heightened post-pandemic pressure in critical-care settings, in line with ICU literature documenting a predominance of MultiDrug-Resistant Organisms (MDROs) and worsened outcomes during the pandemic, and justify evaluation of long-acting, passive interventions (e.g., antimicrobial nanocoatings) as adjuncts infection prevention and control IPC [28,29,30]. Amplifying current AMR mortality concerns, it was estimated that antimicrobial resistance could potentially cause 10 million annual deaths by 2050. Economic implications are substantial, with European healthcare costs associated with antimicrobial resistance exceeding nine billion euros annually, while United States estimate 20 billion dollars for healthcare sector impacts and approximately 35 billion dollars for productivity losses attributed to this antimicrobial resistance crisis [26,27].

### 2.3. One Health Approach and Resistance Mechanisms

Given AMR complexity, multidisciplinary approaches are essential. Combating AMR requires comprehensive “One Health” strategies encompassing human, animal, plant, and environmental health domains. AMR connects all these components through irresponsible and excessive antimicrobial utilization across diverse sectors including agriculture, livestock production, and human medicine [31]. Resistance spread is facilitated by antimicrobial mismanagement, inadequate infection control, agricultural waste, environmental contamination, and migration of people and animals harboring resistant bacteria [31]. Bacteria have developed various adaptive mechanisms conferring antimicrobial agent resistance and ensuring competitive environment survival. Bacterial resistance classification includes different categories [32,33]. Antibiotic-resistant bacteria may possess genes from intrinsic, acquired or adaptive origins. Intrinsic resistance represents bacteria’s inherent natural resistance capacity to specific antibiotic classes through chromosomal gene presence without mutation or additional gene acquisition. Acquired resistance describes evolutionary processes whereby previously sensitive bacteria develop resistance through chromosomal gene mutation or exogenous genetic material acquisition via horizontal gene transfer (HGT). Acquired resistance transmission typically occurs through conjugation-acquired plasmids and may be temporary or permanent. Adaptive resistance represents environmental change-conditional phenotypes that may be interim or permanent depending on selection pressure ability and duration [32,33].

Moreover, it is crucial distinguishing between resistance and persistence. Antimicrobial therapy failure may result from genuinely resistant bacteria or persistent cell presence. Persistent cells develop antimicrobial sensitivity only upon replication resumption, since antibacterials target metabolically active and replicating cells exclusively. The surviving cell fraction, termed ‘persisters’, potentially relates to antibiotic resistance providing viable cell reservoirs enabling resistant mutant emergence through horizontal gene transfer or spontaneous chromosomal mutations [27].

### 2.4. ESKAPE Pathogens

Microbial resistance management faces three primary threats, namely antimicrobial resistance expansion, appropriate therapy absence, and increased biofilm-associated infections [18]. The ESKAPE pathogen group comprises six genera responsible for various nosocomial infections, encompassing both Gram-positive and Gram-negative bacteria. Gram-positive pathogens include major nosocomial organisms such as *Enterococcus faecium* and *Staphylococcus aureus*. *E. faecium* is associated with biofilm-related complications in medical device, capable of producing major bloodstream, surgical site, urinary tract, and central nervous system infections. *S. aureus*, another major opportunistic Gram-positive organism, causes persistent biofilm-associated infections in hospitalized patients. Gram-negative ESKAPE pathogens include *Klebsiella pneumoniae*, *Acinetobacter baumannii*, *Pseudomonas aeruginosa*, and *Enterobacter species*. *K. pneumoniae* forms complex biofilms on various surfaces including medical devices such as catheters and in gastrointestinal mucosa, respiratory and urinary tracts. *A. baumannii* represents an opportunistic pathogen recognized for complex biofilm formation capability, playing critical pathogenesis roles. *P. aeruginosa* participates in chronic cystic fibrosis patient infections and represents a well-established biofilm-forming pathogen providing bacterial protective environments against host defenses and antimicrobial agents [34].

*P. aeruginosa* antibiotic resistance remain challenging due to both high intrinsic resistance and resistance acquisition capability across all antibiotic classes [35]. *Enterobacter species* are commonly found in hospital settings, leading to septicemia, urinary tract infections, post-surgical peritonitis, and pneumonia [34].

Effective AMR management requires engagement from diverse public institutions, stakeholders and healthcare professionals, creating substantial coordination challenges that compound the complexities of international cooperation [36].

## 3. Strategies Adopted in Hospital Settings to Combat Antimicrobial Resistance

### 3.1. International Collaboration and Policy Frameworks

The European public health community has reached broad consensus that international collaboration and coordination represent fundamental components in combating antimicrobial resistance [36].

Global and national antimicrobial resistance control efforts have experienced steady expansion over the previous decade [37]. Two pivotal developments in AMR control include the 2015 World Health Organization global action plan (GAP) on antimicrobial resistance, which mandated all nations to establish national action plans (NAP) by 2017, and the 2016 United Nations political declaration on AMR, incorporating commitments for multisectoral action plan implementation at national, regional, and global levels following One Health principles [37,38]. The 2015 Global Action Plan focused on five primary objectives, and, in particular, on (i) enhancing awareness through educational and training initiatives, (ii) strengthening surveillance and research intelligence, (iii) preventing infections while improving sanitation systems, (iv) optimizing antimicrobial patterns, and (v) establishing economic frameworks for sustainable investment strategies [39,40,41]. European national action plans demonstrated considerable variation in approach from direct Global Action Plan replication to highly independent frameworks. The European Public Health Alliance reports substantial disparities among northern, southern, western, and central-eastern European regions regarding AMR policy implementation effectiveness, particularly concerning financial estimate inclusion, implementation and evaluation mechanism integration, and measurable goal identification [36].

### 3.2. Targets and Classification Systems

The 2024 United Nations political declaration draft on antimicrobial resistance establishes targets for 2030 achievement. The European Union has set antimicrobial utilization targets for 2030, with 2019 serving as the baseline year. These targets include reducing total human antibiotic consumption by 20% and total antibiotic sales for farmed animals and aquaculture by 50%. Additionally, EU-level bloodstream infection reduction targets for 2030 include 15% reduction in methicillin-resistant *Staphylococcus aureus* infections, 10% reduction in third-generation cephalosporin-resistant *Escherichia coli* infections, and 5% reduction in carbapenem-resistant *Klebsiella pneumoniae* infections [37,42].

The World Health Organization “Access, Watch, Reserve (AWARE)” classification system categorizes antibiotics into three distinct groups. Access antibiotics represent generally narrow-spectrum agents with minimal resistance potential, characterized by safety and affordability. Watch antibiotics comprise broad-spectrum agents typically employed for severe infections, associated with high resistance selection pressure and elevated costs. Reserve antibiotics constitute last-resort therapeutic options for treating severe multidrug-resistant infections. Despite increased investment in novel antibiotic development, certain infections remain therapeutically intractable [40,42]. Various antimicrobial compounds have demonstrated in vitro efficacy, though many prove unsuitable for large-scale real-world applications or lack adequate testing [5].

### 3.3. Environmental Control and Cleaning Protocols

Hospital environments have gained recognition as significant contributors to healthcare-associated infections (HAIs) and microorganism transmission, particularly multidrug-resistant organisms (MDROs). Previous research indicates elevated MDRO acquisition risk for patients occupying rooms previously inhabited by infected or colonized individuals. This phenomenon results from nosocomial pathogen survival capabilities on inanimate surfaces for extended periods, inadequate hospital room cleaning between patient occupancies, and potential contaminated surface transmission despite routine cleaning protocols [43]. Intensive care units represent critical infection management settings where patient vulnerabilities and multidrug-resistant microorganism proliferation create substantial care challenges. These units harbor complex microbial community varieties whose biodiversity and clinical implications remain insufficiently understood, contributing to unique setting-specific ecosystems through extensive sanitation protocols, prolonged antimicrobial treatments, and extended recovery periods [44].

### 3.4. Hand Hygiene Protocols and Compliance

Hand hygiene protocols have achieved universal recognition as the single most crucial measure for healthcare-associated infection prevention and antimicrobial resistance control. Comprehensive hand hygiene program implementation encompasses appropriate product and facility provision, healthcare worker education, compliance monitoring, and culture creation. Hospital hand hygiene compliance has consistently proven essential for preventing hospital-induced infections. All healthcare facilities maintain standardized protocols detailing hand hygiene application timing and methodology. However, numerous studies report compliance rates below 50%, facilitating infection transmission between patients and healthcare personnel [45]. The COVID-19 pandemic generated temporary hand hygiene compliance rate modifications and alcohol-based hand sanitizer shortages [46]. Alcohol-based handrub consumption (AHC) and antimicrobial resistance data across 27 Italian hospitals were analyzed during 2017–2021. The findings supported the inverse relationship between AHC and infection rates and antimicrobial-resistant bacteria [47]. Enhanced hand hygiene practices constitute integral infection control strategy components, involving hand cleansing using 60–95% alcohol content hand rubs or soap and water to effectively minimize harmful pathogen presence. As an infection control cornerstone, research demonstrates that effective hand hygiene programs can prevent up to 50% of infections while generating savings up to 16 times implementation costs [48].

### 3.5. Textile Management and Antimicrobial Surfaces

Studies have also demonstrated that healthcare fabrics can harbor substantial microbial burdens. Pathogens can survive for weeks on textiles, persisting even after industrial washing cycles. Textile focus stems from their properties as ideal pathogen substrates, making them difficult to clean using traditional antiseptics except through conventional washing cycles. Additionally, textile movement such as bedsheet and curtain handling can aerosolize fabric-borne pathogens. Current Centers for Disease Control and Prevention guidelines address this risk through appropriate hospital textile washing protocols (collection, sorting, transportation, laundering with proper water temperature, detergent types, disinfectants, rinsing and finishing, drying and ironing), along with disinfection and occasionally sterilization procedures [3].

Environmental cleaning and disinfection protocols have evolved to address persistent environmental contamination challenges with resistant organisms. Traditional cleaning and disinfection methods have been augmented with enhanced environmental cleaning protocols, terminal cleaning procedures for multidrug-resistant infection patient rooms, and occasionally no-touch disinfection technologies including ultraviolet light or hydrogen peroxide vapor systems. Manual cleaning utilizing US Environmental Protection Agency (EPA) registered disinfectants represents widely accepted standard practice. Nevertheless, previous studies suggest cleaning adequacy often proves suboptimal, particularly when focusing exclusively on high-touch surfaces [43].

Enhanced terminal room cleaning following patient discharge, incorporating no-touch technologies such as ultraviolet C (UV-C) or hydrogen peroxide (H_2_O_2_) disinfection, has recently been employed to supplement manual cleaning, increase hospital room environment cleanliness, and reduce important nosocomial pathogen transmission, particularly *Clostridioides difficile* or vancomycin-resistant *enterococci*. UV-C effectiveness in reducing critically important environmental pathogen groups, specifically Gram-negative rods, requires further assessment [43]. Far-ultraviolet-C and direct irradiation below exposure limits represent promising technologies for continuous air and surface decontamination in occupied spaces, though additional studies are necessary to evaluate long-term safety and efficacy [49].

Standardizing manual cleaning quality and ensuring cleanliness presents ongoing challenges [49]. Standard fomite management strategies involve implementing proper disinfection and cleaning practices with periodic environmental cleanliness monitoring. However, cleaned surface recontamination probability remains high. Consequently, antimicrobial surfaces that eliminate or repel pathogens represent additional primary defense lines against pathogen transmission and subsequent infection, introducing infection spread barriers. While conceptually simple, formulating durable antimicrobial coatings with broad-spectrum antimicrobial and antifouling activities has proven challenging. Coatings should demonstrate activity against broad nosocomial pathogen spectrums, exhibit human and environmental non-toxicity, maintain durable sustained activity, and prove effective in real-world applications. Numerous publications report coatings exhibiting antimicrobial potency applicable to high-touch surfaces, desirable for healthcare settings to contribute to Hospital Acquired Infection occurrence reductions [5].

Unfortunately, no specific single standard test exists for assessing acceptable antimicrobial coating efficacy in hospital applications. The optimal available protocol originates from the U.S. Environmental Protection Agency (EPA) recommendations suggest effective products should demonstrate minimum three-log reductions within one-hour post-inoculation compared to control carriers. Limited articles report antimicrobial surface durability regarding simulated UV weathering, disinfectant cleaning, temperature, humidity, or temporal factors. Articles frequently fail to assess coating adhesion across surface type ranges for reasonable applications, potentially influencing coating durability [5].

Continuously active quaternary ammonium disinfectants providing residual antimicrobial activity on undisturbed surfaces for up to 24 h have demonstrated clinically important pathogen recovery reduction in some but not all real-world studies. Although quaternary ammonium-based supplemental coatings reportedly provide prolonged residual efficacy in patient care settings, concerns exist that routine cleaning and disinfection may remove these products. Addressing this concern, the Environmental Protection Agency has recently issued updated guidance requiring efficacy demonstration following multiple abrasion and chemical exposures for supplemental residual antimicrobial coating registration [41].

### 3.6. Antimicrobial Stewardship Programs

HAI prevention represents a priority for reducing patient morbidity, mortality, and costs [20]. The World Health Organization states that improved infection prevention and control (IPC) in healthcare settings represents the most important action for AMR reduction, as this decreases healthcare-associated infections and consequently reduces antimicrobial administration requirements [9].

As increasing numbers of organisms developed antimicrobial agent resistance, standardized antimicrobial prescribing approaches became necessary, resulting in Antimicrobial Stewardship Program development. Antimicrobial Stewardship Programs incorporate healthcare professional education and continuous antimicrobial resistance information dissemination, helping restrict multidrug-resistant organism emergence [50]. Antimicrobial stewardship programs represent organizational measure sets designed to optimize antimicrobial use, improve patient outcomes, reduce AMR and healthcare-associated infections, and decrease healthcare costs. These programs aim to reduce resistance emergence and spread by ensuring healthcare worker adherence to appropriate antimicrobial use protocols (proper dosage, treatment duration, and diagnostic test utilization). Antimicrobial stewardship measures include patient education and screening for determining genuine antibiotic necessity in specific cases, inappropriate antibiotic use reduction, hospital policy updates, high-risk antibiotic use limitation in healthcare settings to reduce antimicrobial resistance risk [40].

### 3.7. Emerging Technologies and Innovation

**Figure 2:**
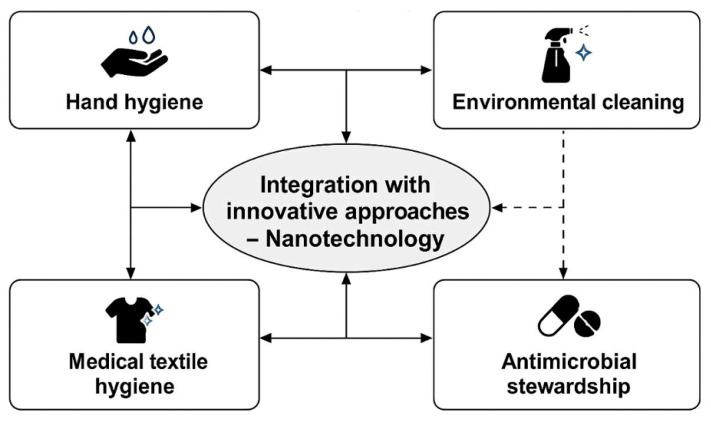
Integrated Infection Prevention and Control (IPC) framework in hospitals and positioning of nanotechnology as a complementary, long-acting barrier.

Emerging technology integration with established infection prevention and antimicrobial stewardship practices represents the next frontier in antimicrobial resistance control. Recognition of traditional limitations has driven interest in innovative approaches that can complement and enhance conventional antimicrobial resistance control strategies. These innovative approaches encompass antimicrobial peptide exploration, bacteriophage therapy, immunomodulatory agents, and nanotechnology-based antimicrobial systems. As summarized in Figure 2, the integrated IPC framework positions nanotechnology as a complementary, long-acting barrier in hospital settings.

## 4. New Antimicrobial Approaches Based on Nanotechnology

### 4.1. Introduction to Nanotechnology in Healthcare

Nanotechnology applications in medical practice encompass diverse therapeutic domains including drug delivery systems, in vitro diagnostic platforms, tissue regeneration protocols, implantable or wearable diagnostic and therapeutic devices, gene therapy applications, dental interventions, oncological treatments, and aesthetic medicine procedures [51,52]. Within healthcare sectors, nanotechnology establishes unprecedented boundaries in life sciences industries, offering exceptional promise for atomic-level manipulation to transform numerous aspects of medical treatment while facilitating sophisticated research instrument development for enhanced therapeutic approaches across various medical conditions [53]. Many advances in the field also highlight the growing potential of green/bio-derived inspired nanotechnology approaches in healthcare applications [54].

### 4.2. Nanotechnology as a Solution to Antimicrobial Resistance

The emergence of nanotechnology as a potential solution for overcoming conventional antimicrobial approach limitations represents a paradigm shift in combating healthcare-associated infections and antimicrobial resistance [51]. Contemporary nanotechnology-based methodologies have garnered substantial attention due to nano-form enhancement of active compounds, providing improved stability and enhanced antimicrobial efficacy compared to identical materials in bulk configurations [55]. Multiple nanomaterial classes, including polymeric systems (natural and synthetic), inorganic nanoparticles (metals and metal oxides), and nano-enabled antimicrobial peptides, provide versatile platforms for developing novel therapeutic strategies [56,57,58]. Among natural polymers, chitosan, alginate, and cellulose derivatives are widely employed owing to their biocompatibility and biodegradability and can enable encapsulation and controlled or stimuli-responsive drug release [59,60,61]. Within synthetic polymers, Poly(lactic-co-glycolic acid) (PLGA) and Polycaprolactone (PCL) offer tunable mechanical properties and programmable degradation kinetics through control of molecular weight, monomer ratios, and crystallinity, whereas Polyethylene glycol (PEG) is commonly used as a matrix component or surface modifier to modulate pharmacokinetic properties in nanomedicines [62,63]. Inorganic nanoparticles can contribute intrinsic antimicrobial activity or act synergistically with polymer carriers, while nano-enabled delivery of antimicrobial peptides enhances stability and pharmacokinetics, collectively broadening the design space for anti-infective interventions [56,57,58].

In addition to their scalability, cost-effectiveness and versatility, these nanomaterials offer advantages including reduced toxicity through lower dosage requirements, diminished resistance development potential, and enhanced antibacterial effects through combined individual action mechanisms [64].

### 4.3. Mechanisms of Action and Biofilm Penetration

Nanoparticles can combat bacteria in both planktonic and biofilm forms using diverse mechanisms, accessing multimodal antibacterial approaches for slowing or preventing drug resistance generation [55,65]. Critical variables affecting biofilm invasion and penetration include size and electrostatic interactions, with nanomaterials smaller than 350 nm demonstrating greater mobility through biofilm pores [66]; moreover, nanomaterial surface charge can enhance interactions with bacterial cells and lipid molecules, increasing bacterial exposure and duration to drugs [67]. The ability of nanoparticles to directly kill bacteria without inducing antimicrobial resistance may enable efficient treatment in cases with limited therapeutic options. Their properties and size facilitate easy bacterial membrane crossing and specific biosynthetic and enzymatic pathway targeting, contrasting with classic antimicrobials that may not enter in adequate concentrations inside target cells due to pore rarity and transport mechanisms required for cell entry [51].

**Figure 3:**
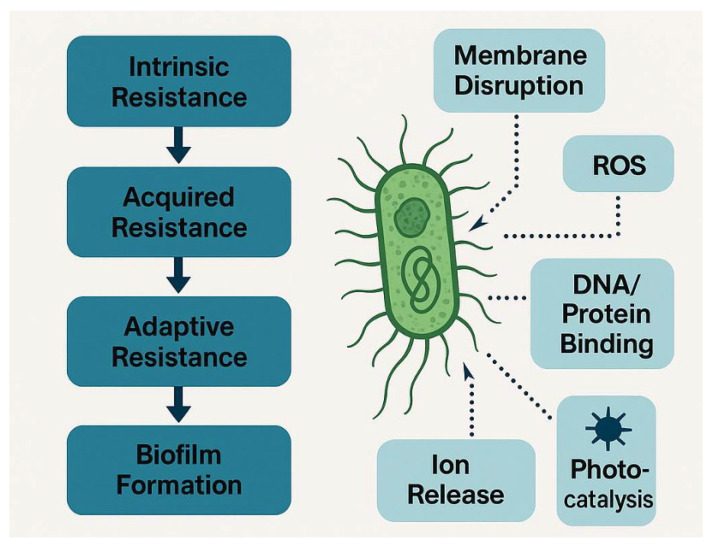
Schematic overview of major resistance mechanisms exhibited by bacteria and multimodal targets of antimicrobial nanomaterials.

A schematic overview of the principal antibacterial mechanisms of metal/metal-oxide nanomaterials, such as membrane perturbation, ion release, ROS-mediated damage, and photo-activated effects is provided in Figure 3, linking each mechanism to representative material classes.

### 4.4. Metal-Based Nanoparticles and Their Efficacy in Resistance Prevention

Metal-based nanoparticles represent the most common inorganic nanoparticles and constitute a promising solution to antibiotic resistance. Various metal nanoparticle types have demonstrated effectiveness as antimicrobial agents, with silver (Ag), gold (Au), copper (Cu), titanium (Ti), nickel (Ni), magnesium (Mg), zinc (Zn), and their oxide-based nanoparticles representing the most commonly utilized antimicrobial nanoagents.

Table 1 provides a quantitative comparison of antimicrobial efficacy among the most promising metallic nanoparticles (silver, gold, copper, zinc) and their oxides, evidencing superior properties by silver nanoparticles and demonstrating the potential of these nanomaterials as next-generation antimicrobial agents against healthcare-associated infections.

Due to their large surface-to-volume ratio, they provide strong, targeted, and prolonged antimicrobial activity with antibiofilm interaction capabilities [85]. They employ action mechanisms that differ significantly from traditional antibiotics. Compared to existing antibiotics, broader antibacterial ranges are demonstrated and, unlike antibiotics where only one bacterial class can be eliminated through single antibacterial mechanisms, different dimensions and shapes can cause death of various exposed bacteria through different mechanisms. These mechanisms include: (i) membrane damage through phospholipid bilayer interactions causing membrane disruption, (ii) cytosolic protein binding and metabolic pathway inhibition, (iii) free radical and reactive oxygen species (ROS) generation for inducing biomacromolecule damage, (iv) DNA intercalation (particularly Ag^+^ ions), (v) enzyme inhibition and ion-mediated stress (well documented for ZnO systems), (vi) photothermal and photocatalytic effects (TiO_2_ under UV light, Cu_2_O under visible light), and (vii) synergistic effects with antibiotics through nano-antibiotic conjugates or co-delivery platforms [65,70,74,85,86,87,88,89,90,91,92].

### 4.5. Historical Context, Modern Applications and Advanced Nanomaterial Categories

Metal materials and metal ions possess extensive antimicrobial use history, with metallic material antibacterial capacities discovered centuries ago. Copper and its compounds were used for treating burns, intestinal worms, ear infections, and other hygienic applications by Greeks, Romans, Aztecs, and other ancient cultures, while silver has been extensively used for medical applications throughout human history. In modern times, nanotechnology utilizes nanosized agent design to optimize conventional drugs, with nanoscale metals demonstrating superior peculiarity in antibacterial applications alongside good safety profiles [93]. Nanoparticle development against troublesome bacteria might represent alternative therapeutic choices with enhanced bactericidal effects and reduced cytotoxicity, with recent years witnessing numerous advances in organic, inorganic, and hybrid nanoparticle applications for combating bacterial infectious diseases [94]. Furthermore, for resistant antimicrobial environments, nanoparticles can be cost-effective and ecological while maintaining flexibility, possessing distinct and well-defined physical and chemical characteristics that can be customized for desirable purposes [95]. Nanoparticles, particularly gold nanoparticles (AuNPs) and silver nanoparticles (AgNPs), demonstrated potential as topical treatments for wound biofilm-associated infections [65] and drug delivery applications. AuNPs can inhibit intracellular adenosine triphosphate (ATP) synthesis and affect transfer RNA binding, thereby enhancing antibiotic permeability to bacterial cells, with studies demonstrating that AuNPs combined with antibiotics significantly improve antibacterial efficacy. AgNPs can effectively penetrate bacterial biofilms and release silver ions to enhance antibacterial activity. Metal nanoparticles can be endowed with various shapes including nanospheres, nanorods, nanostars, nanoshells, and nanocages, while metal oxides such as ZnO, TiO_2_, and SiO_2_ also exhibit abilities to inhibit resistant bacterial strains and prevent biofilm formation [96]. Beyond traditional metal nanoparticles, advanced nano-platforms offer unique advantages. Hybrid/composite systems such as Ag-chitosan, Ag-TiO_2_, and Cu-doped ZnO often show improved stability and, in several models, attenuated potential cytotoxicity relative to free metals/oxides, while retaining or enhancing antimicrobial potency through capping/immobilization and tunable ion release [97,98,99]. Liposomal vancomycin and other lipid-based carriers enable more targeted antimicrobial delivery with lower systemic exposure/toxicity and improved pharmacokinetics, whereas polymeric nanoparticles provide controlled release and can enhance bioavailability of anti-infectives [100,101]. Graphene oxide and MXenes have also emerged as 2D antimicrobial materials with notable antibacterial activity, leveraging high specific surface area and tunable surface chemistry, and showing promise as stand-alone agents or supports in hybrid constructs [102,103].

### 4.6. Surface Modification and Medical Device Applications

Numerous efforts have been made to utilize antibacterial nanoparticles for surface modification through different artificial antibacterial surface fabrications, including surface functionalization, derivatization, polymerization, and mechanical architecture modification, enabling medical devices and implants to resist bacterial adhesion and inhibit bacterial proliferation, reducing surgery or implant-associated hospital-acquired infection risks [93]. Hundreds of different biomaterial surface modifications have been described to equip biomaterial surfaces with antimicrobial properties, and common modifications include cationic coatings, silver nanoparticle coatings and antibiotic-releasing coatings [104].

Antimicrobial-releasing coatings have undergone the most research for preventing medical implant-linked infections, with the search for innovative anti-infective biomaterials raising reasonable hope for averting infection problems connected to both long-term medical implant use and surgical treatment [105].

Nanoparticles (NPs) have been deposited on medically relevant surfaces, medical devices, and implants through techniques including layer-by-layer assembly, sonochemistry, and spin coating to engineer durable nanostructured coatings against biofilm formation [55].

### 4.7. Safety Considerations and Regulatory Aspects

While nanomaterials offer therapeutic benefits, cytotoxicity to human cells (e.g., lung, skin, renal) is dose- and material-dependent, so an appropriate therapeutic window must be defined to maximize antimicrobial efficacy while minimizing host–cell damage. Recent work underscores the roles of dose, size, surface chemistry and ion release in shaping toxicity and the need for safe-by-design optimization [106,107,108]. Ecotoxicity considerations include the release/leaching of nanoparticles into wastewater systems, with potential effects on microbial communities and treatment performance, highlighting the importance of fate and transport assessments for sustainable implementation [109].

From a regulatory perspective, devices incorporating nanomaterials fall under EU MDR (Reg. 2017/745; Rule 19). For antimicrobial surface performance, internationally used laboratory methods include ISO 22196 (antibacterial activity on non-porous surfaces) and ISO 21702 (antiviral activity on non-porous surfaces) [110,111].

## 5. Silver Nanoparticles as Innovative Solutions for HAI Control in Clinical Settings

### 5.1. Historical Foundation and Antimicrobial Mechanisms

Metal silver, silver salts, and silver nanoparticles (AgNPs) possess an extensive historical foundation in preventing and treating infectious diseases, with their antimicrobial applications traceable to ancient time. The medicinal utilization of silver originated when Credé first published in 1881 that silver could effectively treat and cure neonatal eye infections in Germany. Subsequently, in 1933, Gorden and colleagues demonstrated silver’s antimicrobial activity against dental infections [85]. While precise antimicrobial mechanisms remain incompletely elucidated, current studies suggest direct toxic effects by AgNPs on bacterial cell walls combined with silver ion (Ag^+^) release capable of disrupting cell membranes and various intracellular functions. These antimicrobial mechanisms confer broad-spectrum activity against viruses, bacteria, fungi, and parasites while maintaining a high resistance barrier [112]. The established antimicrobial mechanism of AgNPs encompasses DNA replication inactivation, cellular protein denaturation, cell membrane damage, and generation of free radicals including reactive oxygen species derived from nanoparticle surfaces that damage biomacromolecules [85].

Contemporary research has established that silver nanoparticles demonstrate superior advantages over metallic silver due to their prolonged silver ion release capabilities, enhanced surface-to-volume ratio, reduced susceptibility to sequestration by competing ions such as chloride and phosphate, minimal risk of bacterial resistance development, and exceptional antifouling and antibiofilm potential. These characteristics have positioned AgNPs as optimal candidate materials for medical device coatings aimed at preventing healthcare-associated infections caused by extensive pathogenic biofilm networks formed on medical devices [85], with multiple factors including nanoparticle size, shape, surface chemistry, and functionalization influencing the antimicrobial efficiency of the nanoparticle-based coatings [113].

### 5.2. Broad-Spectrum Antimicrobial Activity

**Figure 4:**
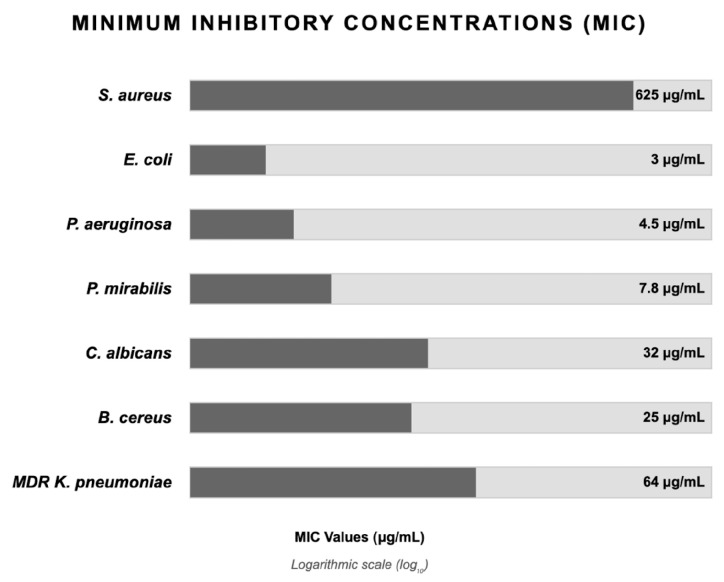
Minimum inhibitory concentration values reported in the literature for silver nanoparticles [114,115,116,117,118,119,120].

Silver in ionic or nanoparticle forms exhibits broad-spectrum antimicrobial activity against biofilm-forming pathogenic microorganisms including *Pseudomonas aeruginosa*, *Staphylococcus epidermidis*, methicillin-resistant *Staphylococcus aureus* (MRSA), and methicillin-resistant *S. epidermidis* (MRSE) strains, along with numerous other microbial colonizers associated with biomedical infections [85]. Figure 4 collects the values of minimum inhibitory concentrations (MIC) reported in the literature for silver nanoparticles against different microorganisms [114,115,116,117,118,119,120].

### 5.3. Recent Advances in Clinical Applications

**Figure 5:**
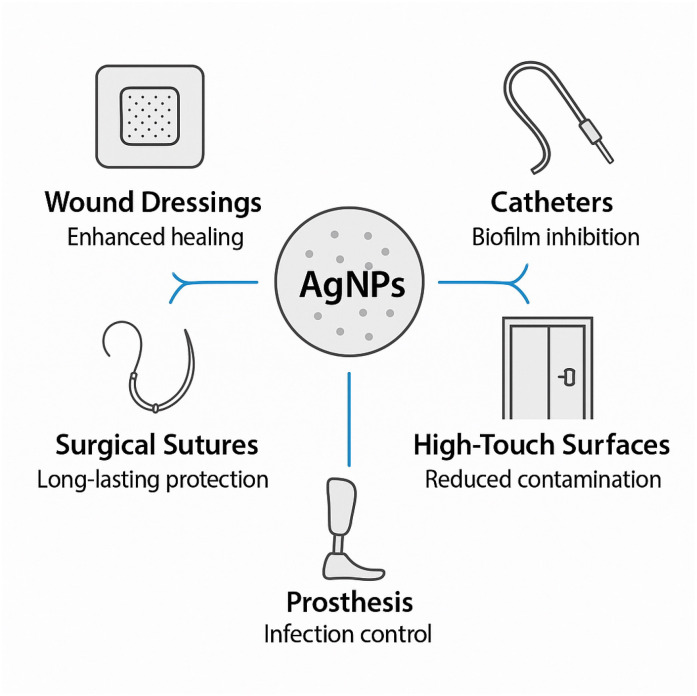
Examples of clinical applications of silver nanoparticles: wound dressings, catheters, surgical sutures, and high-touch hospital surfaces.

Clinically relevant use-cases for antimicrobial nano-silver include wound dressings, urinary devices, environmental surface coatings, and hospital textiles, highlighting how these interventions integrate with standard infection prevention and control practices (Figure 5).

#### 5.3.1. Wound Care Applications

AgNP antimicrobials are predominantly suggested as topical agents where they are incorporated into diverse wound dressing formulations, demonstrating enhanced wound healing promotion capabilities. The small size and large surface area of dressing-integrated AgNPs enhance solubility, promote high-concentration wound coverage, and facilitate Ag^+^ release in low non-toxic and sustained concentrations [112]. Recent clinical evidence demonstrates that wound dressing applications based on silver-based products have been patented and commercialized with increasingly complex designs and improved efficacy compared to standard dressings [121]. Biopolymers combined with bioactive antimicrobial, antibacterial, and anti-inflammatory nanoparticles demonstrate exceptional potential in wound care for promoting healing, particularly in managing diabetic foot ulcers, which continue to represent enormous clinical challenges associated with high amputation rates and substantial healthcare costs [122]. The unique AgNPs properties suggest dual functionality in effectively preventing wound infections while improving damaged tissue healing processes [123].

Clinical investigations have demonstrated that severe infected wounds will likely be treated with combined therapy utilizing AgNPs and standard antibiotics in the near future. The effectiveness of such combination therapies has been validated in studies exploring dressings combining silver with colistin or neomycin [124]. Several natural or synthetic biomaterials containing silver nanoparticles have presented promising results in pre-clinical and clinical trials, including the multicenter, prospective randomized controlled VULCAN study performed on 213 patients with venous leg ulceration to evaluate silver dressing effectiveness compared to non-antimicrobial non-adherent dressings [125].

#### 5.3.2. Medical Device Applications

AgNP-based coatings on medical devices have been extensively investigated on substrates including polyurethane catheters for antibacterial and antibiofilm applications. Substantial research has focused on AgNP-fabricated catheters and their role in preventing catheter-associated urinary tract infections (CAUTIs) or catheter-related bloodstream infections (CBSIs). AgNP-coated urinary catheters demonstrate superior benefits compared to conventional catheters, with significantly reduced CAUTI rates following silver nanocoating introduction, and absolute risk reduction values for catheter-associated bacteria ranging from 0.5% to 32% [85,126]. AgNPs coated catheters had a significant inhibitory effect on the colonization of the devices by antibiotic-resistant *Staphylococcus epidermidis* and *Staphylococcus aureus*, *Escherichia coli*, *Klebsiella pneumoniae*, *Proteus mirabilis*, and *Pseudomonas aeruginosa* [127].

Silver nanoparticle coating technology has also revolutionized surgical suture applications through multiple innovative approaches. AgNPs have been successfully deposited onto silk surgical sutures using environmentally friendly synthesis methods, demonstrating exceptional antimicrobial characteristics and biocompatibility. The coated sutures maintain biocompatibility through 3-(4,5-dimethylthiazol-2-yl)-2,5-diphenyltetrazolium bromide (MTT) assays using 3T3 fibroblast cell cultures while demonstrating prolonged antimicrobial activity [128]. Research studies have validated the effectiveness of propolis-silver nanoparticle combinations for surgical suture coating, with in vitro antimicrobial assays demonstrating significant activity against clinical pathogenic microorganisms, supporting combined propolis and silver nanoparticle synergistic effects for clinical practice and biomedical applications [129].

#### 5.3.3. Hospital Surface Applications

Beyond medical devices, high-contact surfaces in healthcare facilities, including door handles, light switches, and elevator controls, function as pathogen transmission hotspots. Silver nanoparticle-based antimicrobial coatings applied to these surfaces significantly reduce microbial loads and enhance cleaner and safer environments. Antimicrobial surface coatings minimize infection transmission among patients, healthcare staff and visitors, particularly in high-risk environments such as emergency rooms and operating theaters. Some investigations have demonstrated the effectiveness of antimicrobial nanocoatings in medical environments, assessing that silver nanoparticle-coated hospital surfaces significantly reduce bacterial contamination [130,131,132,133]. A landmark clinical study by Ellingson and colleagues investigated antimicrobial surface coating impacts on HAIs and environmental bioburdens at two urban hospitals. Transparent antimicrobial surface coatings were applied to patient rooms and common areas in three units at each hospital. Following surface application, total bacterial colony-forming units declined by 79% and 75% at the two hospitals, with statistically significant reductions in HAIs and environmental bioburdens in units receiving the antimicrobial surface coating [134].

#### 5.3.4. Clinical Trials and Ongoing Research

Several EU-supported programs are advancing durable, safe-by-design antimicrobial nanocoatings towards real-world use in healthcare environments (e.g., NOVA [135], MIRIA [136,137], STOP [138,139] projects. These projects focus on high-touch clinical surfaces, textiles and equipment, emphasize sustainability and standardized performance testing, and include pilot-scale validations in relevant settings. Complementing these efforts, a double-blind, placebo-controlled randomized trial conducted in a hospital Emergency Department demonstrated significant short-term reductions in surface bacterial burden on coated high-touch sites, supporting clinical feasibility for routine care environments [140]. In orthopedics, silver-containing surface coatings, including silver-containing hydroxyapatite (Ag-HA) and silver-coated megaprostheses—have already entered clinical practice [141,142]. Observational series report lower infection rates or improved infection control in selected indications, and randomized trials are being designed to rigorously assess clinical benefit and safety [143].

Examples of clinical and near-clinical applications of antimicrobial silver-based nanomaterials pertinent to healthcare-associated infection prevention and control are collected in Table 2 for wound dressings, urinary catheters, surgical sutures, environmental surface coatings, central venous catheters, and hospital textiles. For each application area, the table reports the clinical context, a brief evidence synthesis, the observed or expected clinical impact.

The combination of antimicrobial efficacy, biocompatibility at therapeutic concentrations, and multiple simultaneous action mechanisms address silver nanoparticles as innovative solutions for healthcare-associated infection control across diverse clinical applications, from advanced medical devices to hospital surface decontamination systems, thus providing evidence of the successful silver-based antimicrobial approach. Together, these developments illustrate a maturing translational pipeline from preclinical validation to controlled clinical evaluation for antimicrobial coating technologies.

## 6. Conclusions and Future Perspectives

The comprehensive analysis presented in this review demonstrates that healthcare-associated infections remain an important challenge in contemporary medical practice, with their impact substantially amplified by the growing threat of antimicrobial resistance. The convergence of these two critical issues has created an urgent imperative for innovative solutions that overcome the limitations of conventional antimicrobial approaches.

Scientific evidence supports the big potential of nanotechnology, particularly silver nanoparticles, as a valuable approach to healthcare-associated infection prevention and control. Unlike traditional antimicrobial agents that target single pathways and are susceptible to resistance mechanisms, silver nanoparticles demonstrate multiple simultaneous mechanisms of action, which significantly reduce the likelihood of resistance development while providing broad-spectrum efficacy against bacteria, fungi, viruses, and parasites. The application of silver nanoparticle coatings to hospital surfaces represents a revolutionary approach to environmental infection control. Results from clinical studies suggest that comprehensive surface coating strategies could fundamentally alter the infection landscape in hospitals, potentially preventing thousands of infections annually with significant economic implications. The potential savings from prevented infections, reduced antibiotic use, shortened hospital stays, and decreased morbidity and mortality could result in significant net cost reductions for healthcare systems.

Nanotechnology-based antimicrobial systems should function in conjunction with, rather than as replacement for, current infection prevention and control (IPC) practices. The optimal approach involves integrating nanomaterial applications with established hand hygiene protocols, environmental cleaning procedures, and antimicrobial stewardship programs leveraging complementary strengths while mitigating individual limitations. However, despite promising results, some limitations must be acknowledged.

Regarding cost and scalability concerns, high-quality nanoparticle manufacturing still faces scale-up/quality challenges, which keep unit costs elevated; nonetheless, economic evaluations show that IPC interventions are generally cost-effective, suggesting that investments in validated adjunct technologies may be justified when they prevent HAIs [156,157]. In terms of long-term efficacy questions, the durability of antimicrobial coatings must be verified under repeated cleaning, mechanical stress, and variable environments. Evidence includes a double-blind randomized controlled trial (RCT) in an Emergency Department showing 24 h reduction in surface bioburden, laboratory studies showing retained activity after bleach cleaning, and reviews stressing the need for cleaning-resistant coatings and more realistic test methods [158,159]. The regulatory pathway for nanomaterial-based medical devices also remains complex and evolving, and harmonized international standards are needed to facilitate clinical translation while ensuring patient safety.

Future research priorities should include (i) development/uptake of standardized efficacy protocols for antimicrobial surfaces and realistic exposure tests; long-term safety studies in diverse patient populations; (iii) economic impact evaluations across settings; (iv) environmental studies integrated with risk assessment; and (v) regulatory harmonization to streamline translation while ensuring safety [110,159,160].

Standardization of manufacturing processes, optimization of coating methodologies, along with establishment of comprehensive safety profiles require continued research and development efforts. The transition from laboratory research to clinical implementation requires coordinated efforts among researchers, clinicians and industry partners. Successful implementation requires collaboration among material scientists, clinicians, microbiologists, regulatory experts, and healthcare administrators. This approach ensures that technological innovations address real clinical needs while meeting safety and efficacy standards. Success will depend on multidisciplinary collaboration, sustainable implementation strategies, and continued innovation in nanomaterial design and application.

## Figures and Tables

**Table 1:** Comparison of the antimicrobial efficacy demonstrated by the most effective nanomaterials.

Nanomaterial	Size (nm)	Tested Pathogens	Antimicrobial Efficacy %	Mechanisms of Action	References
**Silver Nanoparticles** **(AgNPs)**	2–100	*S. aureus*, *E. coli*, *P. aeruginosa*, *A. flavus*, *C. albicans*	95; >99.9% bacterial reduction and biofilm disruption	Cell membrane disruption, Ag^+^ release, ROS generation, DNA binding, protein synthesis inhibition, cell division arrest	[34,65,68,69,70,71,72]
**Zinc Oxide** **(ZnO)**	21–100	*S. aureus, P. aeruginosa, E. coli, S. epidermidis, B. subtilis*	80–92% bacterial reduction and growth inhibition	Photocatalysis, ROS generation (•OH, O_2_^−^, H_2_O_2_), Zn^2+^ release, cell wall disruption, intracellular pH alteration	[73,74,75,76]
**Copper-based Nanoparticles (CuNPs/CuO)**	4.5–40	*S. aureus, E. coli, P. aeruginosa, B. subtilis*	85–95% bacterial reduction and killing efficacy	Cu^2+^ ion release, ROS generation, membrane disruption, intracellular oxidative stress, DNA damage, respiratory enzyme inhibition, cytoplasmic protein denaturation	[77,78,79,80,81]
**Gold Nanoparticles (AuNPs)**	5–15	*S. aureus*, *E. coli*, *MRSA*	65–85% bacterial reduction (synergistic with antibiotics)	ATP synthesis inhibition, protein binding, tRNA binding interference, increased membrane permeability to antibiotics, enhanced antibiotic uptake, membrane destabilization	[65,67,82,83,84]

**Table 2:** Recent advances in silver nanoparticle applications for healthcare-associated infection control.

Application Area	Specific Use	Key Findings	Clinical Impact	References
Wound Dressings	Diabetic foot ulcers; surgical wounds	Improved healing rates and reduced bioburden (clinical studies/meta-analyses)	Shorter time-to-healing in selected indications	[144,145,146]
Urinary Catheters	CAUTI prevention	Risk reduction of bacteriuria/CAUTI with silver-alloy or noble-metal-alloy catheters (setting-dependent)	Significant CAUTI reduction vs. standard catheters; effect varies by duration/material	[147,148,149]
Surgical Sutures	Post-operative infection prevention	Sustained antimicrobial activity (preclinical evidence)	Clinical benefit shown for silver dressings on surgical incisions	[150,151]
Hospital Surfaces	High-touch surface coating	75–79% reduction in environmental contamination (study-dependent)	Decreased HAI transmission in multi-unit studies	[134,140,152]
Central Venous Catheters	CLABSI prevention	Reduced catheter colonization/CRBSI with antimicrobial-impregnated CVCs	Improved outcomes in meta-analyses	[153]
Textile Applications	Hospital linens; uniforms	Persistent antimicrobial activity retained after washing (technology-dependent)	Potential IPC benefit via reduced textile contamination	[154,155]

## Abbreviations

HAIs	Healthcare-associated infections
AMR	Antimicrobial resistance
CLABSIs	Central line-associated bloodstream infections
CAUTIs	Catheter-associated urinary tract infections
SSIs	Surgical site infections
VAP	Ventilator-associated pneumonia
MRSA	Methicillin-resistant Staphylococcus aureus
HIV	Human Immunodeficiency Virus
GLASS	Global Antimicrobial Resistance and Use Surveillance System
HGT	Horizontal Gene Transfer
GAP	Global Action Plan
NAP	National Action Plans
AHC	Alcohol-Based Handrub Consumption
MDROs	Multidrug-Resistant Organisms
EPA	Environmental Protection Agency
